# Lying-In-Hospitals

**Published:** 1888-03-31

**Authors:** 


### The Hospital

A Weekly Institutional Journal of

**Science, Medicine, Nursing, and Philanthropy.**

*For Hospitals, Asylums, Medical Practitioners, Students, Nurses, Families, Parishes, Congregations, and the Charitable Public.*

Vol. III. No. 79.] [March 31, 1888.

### Lying-in Hospitals.

Among the numerous charities around us courting public favour, there are none that appeal more directly to our instincts of manhood than those which have been established as homes for women in their hour of trial. Our ancestors, fully alive to their duties to the weaker sex, early turned their sympathetic attention to the necessity of making provision for lonely and helpless women during their confinement, and maternity hospitals were established in the large towns throughout the country soon after the general hospitals were set going. The renaissance of hospitals in this country, after the Reformation, dates from the first and second quarters of the last century; and about the middle of the century no fewer than four lying-in hospitals and an extern maternity were founded in London alone, while the general hospitals, excluding the monastic foundations, might have been counted on the fingers of one hand. In those days special hospitals as we now know them could scarcely be said to have existed. It is true that houses for lepers dragged on their operations well into the century, but leprosy was gradually being stamped out of the country, and the miserable buildings in which the unfortunates found refuge were being demolished. The only curative institution in London, apart from the lying-in hospitals, which could be dignified by the name of special, was the smallpox hospital, instituted in 1746, and mainly for the purposes of inoculating the disease. The original lying-in hospitals long held their own against all claimants for public sympathy, but it is very strange that from that date to this there has been no addition made to the number of these meritorious institutions, while curative establishments of every other conceivable character have sprung into being to the number of a hundred and more, in the metropolis alone. It is not difficult for the initiated to assign a cause for this apparent neglect of institutions which our forefathers thought so well of. It was soon discovered that more women died from the effects of child-birth in these establishments than was the case at their own homes, and the mortality at times became so excessive that it was found necessary periodically to close them in order that they might undergo a kind of forced quarantine. Even in times as remote as the last century, when little attention was paid to medical statistics, the facts were commented on by the leading physicians of the day, but no remedial measures practised at the time were of any avail. If we are to believe Mr. Ryan, who is well entitled to be heard from his experience of Queen Charlotte's Hospital, this high mortality, with occasional intermissions, has continued till a very recent period (1879), but during the past eight years matters have improved much and there is a reasonable prospect of better result sin future. The death-rate has declined from ninetee nto eleven per thousand women confined in the three lying-in hospitals, which he quotes, and he tells us that it would have been reduced still further had the City of London Hospital in the City Road not been included. Although we hail with satisfaction this turn in the tide, it is greatly to be feared that the future experience of a hundred and fifty years will continue to mar the history of these charities. Nor can it be said that the present death-rate in these establishments, although vastly more favourable than the records of former times, is much to boast of. That one woman out of every hundred should die in giving birth to children is a higher mortality than, after all that may be said to the contrary, ought to be the case in what is but a healthy natural process; and until lying-in hospitals are able to show a record which can compare with, or even approach the standard, of the Registrar-General in his statistics of child-births, few sanitary authorities would give their sanction to the extension of these charities unless to the homes of the patients. Dr. Farr in his analysis of twenty-eight years of the death-rate in child-birth in England and Wales made it clear that the deaths in the case of those confined at home, did not exceed one in two hundred, and the records of extern maternity charities all go to confirm this opinion. On the other hand, lying-in hospitals are more serviceable as schools of instruction for medical men, midwives, and monthly nurses, and every effort should be made to diminish the risks to the poor women who flock to them as havens of refuge. Much has been done in the general hospitals to stamp out septic disease from surgical wards, and we have it on the authority of many medical men as well as of Mr. Ryan that since the introduction of the antiseptic system into midwifery hospitals, the mortality has been materially reduced. There is no doubt that puerperal septicæmia is one of the most infectious forms of all spreading distempers, and as no class of mothers is exempt from its ravages, too much caution cannot be employed to guard against its supervention in all labours. As far as we know, the pavilion cubicle system of hospital construction has not been introduced into the midwifery hospitals of this country; but this principle of isolation has been acted on elsewhere, and we are informed with the best results. Nor is there any reason why, in spite of the unsatisfactory experience of King's College Hospital, an intern obstetric department should not be attached to each of our great hospitals where students and nurses could be practically taught without having to emigrate to the Rotunda or to sojourn for a while in a lying-in hospital nearer home. It is true that each hospital associated with a medical school possesses a gynæcological, more frequently called "obstetric" department, but it has no connexion with the latter except in name, and the student is left to pick up a knowledge of his profession by means of a maternity outside. It would be an obvious benefit to all if such an arrangement could be initiated with safety, but until then we must rest under the opprobrium that the septic conditions, which periodically are apparently inseparable from parturition when conducted in lying-in hospitals, are insurmountable.

